# Apathy in presymptomatic genetic frontotemporal dementia predicts cognitive decline and is driven by structural brain changes

**DOI:** 10.1002/alz.12252

**Published:** 2020-12-14

**Authors:** Maura Malpetti, P. Simon Jones, Kamen A. Tsvetanov, Timothy Rittman, John C. van Swieten, Barbara Borroni, Raquel Sanchez‐Valle, Fermin Moreno, Robert Laforce, Caroline Graff, Matthis Synofzik, Daniela Galimberti, Mario Masellis, Maria Carmela Tartaglia, Elizabeth Finger, Rik Vandenberghe, Alexandre de Mendonça, Fabrizio Tagliavini, Isabel Santana, Simon Ducharme, Chris R. Butler, Alexander Gerhard, Johannes Levin, Adrian Danek, Markus Otto, Giovanni B. Frisoni, Roberta Ghidoni, Sandro Sorbi, Carolin Heller, Emily G. Todd, Martina Bocchetta, David M. Cash, Rhian S. Convery, Georgia Peakman, Katrina M. Moore, Jonathan D. Rohrer, Rogier A. Kievit, James B. Rowe, Genetic FTD Initiative (GENFI)

**Affiliations:** ^1^ Department of Clinical Neurosciences Cambridge University Hospitals NHS Trust University of Cambridge Cambridge UK; ^2^ Department of Neurology Erasmus Medical Centre Rotterdam Netherlands; ^3^ Department of Clinical and Experimental Sciences Centre for Neurodegenerative Disorders University of Brescia Brescia Italy; ^4^ Alzheimer's disease and Other Cognitive Disorders Unit Neurology Service Hospital Clínic Institut d'Investigacións Biomèdiques August Pi I Sunyer University of Barcelona Barcelona Spain; ^5^ Department of Neurology Cognitive Disorders Unit Donostia Universitary Hospital San Sebastian Spain; ^6^ Neuroscience Area Biodonostia Health Research Institute San Sebastian Gipuzkoa Spain; ^7^ Clinique Interdisciplinaire de Mémoire Département des Sciences Neurologiques CHU de Québec Faculté de Médecine Université Laval Québec Canada; ^8^ Department of Neurobiology Care Sciences and Society Center for Alzheimer Research Division of Neurogeriatrics Bioclinicum Karolinska Institutet Solna Sweden; ^9^ Unit for Hereditary Dementias Theme Aging Karolinska University Hospital Solna Sweden; ^10^ Department of Neurodegenerative Diseases Hertie‐Institute for Clinical Brain Research and Center of Neurology University of Tübingen Tübingen Germany; ^11^ Center for Neurodegenerative Diseases (DZNE) Tübingen Germany; ^12^ Fondazione Ca’ Granda IRCCS Ospedale Policlinico Milan Italy; ^13^ Centro Dino Ferrari University of Milan Milan Italy; ^14^ Sunnybrook Health Sciences Centre Sunnybrook Research Institute University of Toronto Toronto Canada; ^15^ Tanz Centre for Research in Neurodegenerative Diseases University of Toronto Toronto Ontario Canada; ^16^ Department of Clinical Neurological Sciences University of Western Ontario London Ontario Canada; ^17^ Department of Neurosciences Laboratory for Cognitive Neurology KU Leuven Leuven Belgium; ^18^ Neurology Service University Hospitals Leuven Leuven Belgium; ^19^ KU Leuven Leuven Brain Institute Leuven Belgium; ^20^ Faculty of Medicine University of Lisbon Lisbon Portugal; ^21^ Fondazione IRCCS Istituto Neurologico Carlo Besta Milano Italy; ^22^ University Hospital of Coimbra (HUC) Neurology Service Faculty of Medicine University of Coimbra Coimbra Portugal; ^23^ Center for Neuroscience and Cell Biology Faculty of Medicine University of Coimbra Coimbra Portugal; ^24^ Department of Psychiatry McGill University Health Centre McGill University Montreal Québec Canada; ^25^ McConnell Brain Imaging Centre Montreal Neurological Institute McGill University Montreal Québec Canada; ^26^ Nuffield Department of Clinical Neurosciences Medical Sciences Division University of Oxford Oxford UK; ^27^ Division of Neuroscience and Experimental Psychology Wolfson Molecular Imaging Centre University of Manchester Manchester UK; ^28^ Departments of Geriatric Medicine and Nuclear Medicine University of Duisburg‐ Essen Duisburg Germany; ^29^ Department of Neurology Ludwig‐Maximilians Universität München Munich Germany; ^30^ German Center for Neurodegenerative Diseases (DZNE) Munich Germany; ^31^ Munich Cluster of Systems Neurology (SyNergy) Munich Germany; ^32^ Department of Neurology University of Ulm Ulm Germany; ^33^ IRCCS Centro San Giovanni di Dio Fatebenefratelli Brescia Italy; ^34^ Molecular Markers Laboratory IRCCS Istituto Centro San Giovanni di Dio Fatebenefratelli Brescia Italy; ^35^ Department of Neuroscience Psychology Drug Research and Child Health University of Florence Florence Italy; ^36^ IRCCS Fondazione Don Carlo Gnocchi Florence Italy; ^37^ Department of Neurodegenerative Disease Dementia Research Centre UCL Queen Square Institute of Neurology University College London London UK; ^38^ MRC Cognition and Brain Sciences Unit University of Cambridge Cambridge UK; ^39^ Cognitive Neuroscience Department Donders Institute for Brain Cognition and Behavior Radboud University Medical Center Nijmegen Netherlands

**Keywords:** apathy, cognitive decline, genetic frontotemporal dementia, longitudinal design, MRI, presymptomatic carriers

## Abstract

**Introduction:**

Apathy adversely affects prognosis and survival of patients with frontotemporal dementia (FTD). We test whether apathy develops in presymptomatic genetic FTD, and is associated with cognitive decline and brain atrophy.

**Methods:**

Presymptomatic carriers of *MAPT*, *GRN* or *C9orf72* mutations (N = 304), and relatives without mutations (N = 296) underwent clinical assessments and MRI at baseline, and annually for 2 years. Longitudinal changes in apathy, cognition, gray matter volumes, and their relationships were analyzed with latent growth curve modeling.

**Results:**

Apathy severity increased over time in presymptomatic carriers, but not in non‐carriers. In presymptomatic carriers, baseline apathy predicted cognitive decline over two years, but not vice versa. Apathy progression was associated with baseline low gray matter volume in frontal and cingulate regions.

**Discussion:**

Apathy is an early marker of FTD‐related changes and predicts a subsequent subclinical deterioration of cognition before dementia onset. Apathy may be a modifiable factor in those at risk of FTD.

## 1 INTRODUCTION

Apathy is a common and disabling feature of frontotemporal dementia (FTD). It is part of the diagnostic criteria for behavioral variant of FTD (bvFTD),^1^ and frequently occurs across all FTD variants.^2^, ^3^ Apathy is a multifaceted construct that describes dysfunctional goal‐directed behavior, arising from affective, behavioral, and cognitive impairments. FTD has been associated with concurrent affective, behavioral, and cognitive apathy symptoms,^4^ which worsen the prognosis in terms of survival,^5^ disability^6^, ^7^, ^8^, ^9^ and functional independence. Better understanding of the causes and consequences of apathy and its role in the clinical progression of FTD is vital to develop effective treatment strategies, including preventive strategies in the context of genetic risk of FTD.

Previous imaging studies have identified structural correlates and changes associated with apathy in FTD. The severity of apathy correlates with widespread atrophy in frontotemporal areas, including the dorsolateral, ventromedial and orbital prefrontal cortex, anterior cingulate cortex, and insula and basal ganglia^3^, ^10^, ^11^, ^12^ (see^13^, ^14^). In people with symptomatic FTD, apathy is associated with the severity of executive function impairment,^12^, ^15^ including deficits in working memory, decision making, selective/sustained attention, planning, processing speed, inhibitory processes and mental/cognitive flexibility.^12^, ^15^, ^16^, ^17^, ^18^ Deficits in executive function occur in both behavioral and aphasic syndromes of FTD, with subtler impairments in the presymptomatic phase.^19^, ^20^, ^21^ Indeed, executive dysfunction, like apathy, is a diagnostic criterion for bvFTD^1^ and shares several anatomical correlates with apathy (see ^13^ for a review). Although no single task captures all domains and processes associated with executive function, there are commonly used tasks that encompass relevant cognitive processes to provide sensitive markers for executive function. For example, the Digit Symbol Substitution test of the Wechsler Adult Intelligence Scale–Revised (WAIS‐R) depends on a combination of the components of executive function (working memory, attentional control, and rule sets), in addition to non‐executive visuospatial domains and processing speed.^22^, ^23^ The Digit Symbol test correlates with other measures of executive function and is sensitive to the presence of cognitive changes in patients with frontal lobe damage and dementia.^20^, ^24^, ^25^, ^26^, ^27^, ^28^ We therefore use the Digit Symbol test performance as an index of executive dysfunction in presymptomatic FTD.

The causal relationship between apathy and executive dysfunction in FTD remains unclear: specifically, whether apathy predicts cognitive decline, or vice versa. This is especially relevant to the emergence of FTD symptoms in those at genetic risk. A third of patients with FTD present an autosomal dominant family history,^29^ with mutations of three main genes accounting for about a fifth of cases: microtubule‐associated protein tau (*MAPT*), progranulin (*GRN*), and chromosome 9 open reading frame 72 (*C9orf72*).^29^, ^30^ We therefore examined longitudinal changes in apathy and their association with subclinical cognitive decline in presymptomatic gene carriers, in the international Genetic FTD Initiative (GENFI).^20^

We first tested the hypothesis that apathy increases over time in presymptomatic carriers of FTD mutations, and is more severe in those closer to symptom onset. We used latent growth curve modeling of longitudinal data to test the predictive value of apathy for subclinical deterioration of cognitive performance in the Digit Symbol test in gene carriers versus non‐carriers. To understand the relationship between apathy and FTD‐related brain changes, we tested whether baseline and longitudinal changes in apathy were a function of atrophy in the presymptomatic gene carriers. Previous studies suggest a detrimental effect of apathy on clinical progression and survival of FTD patients,^5^, ^6^, ^7^, ^8^, ^9^ and have highlighted frontal lobe and cingulate cortex atrophy as neural correlates of apathy in FTD.^13^, ^14^ Based on this, we predicted: (1) that baseline apathy predicts future cognitive deterioration; and (2) an association between apathy and structural brain change, in the frontal lobe and cingulate cortex.

## 2 METHODS

### 2.1 Participants

From the GENFI study,^20^ DataFreeze 4 (2019), 600 participants were included in this study: 304 presymptomatic mutation carriers (54 with mutation in *MAPT*, 142 in *GRN*, and 108 in *C9orf72*), and 296 family members without mutations (non‐carrier control group). To meet the inclusion criteria, all participants needed to not present another significant medical or psychiatric condition that would interfere in their completion of assessments or impair their safety in the study. Participants in pregnancy, or with contraindications to MRI were not recruited.

**Highlights**

- Apathy progresses in presymptomatic genetic frontotemporal dementia
- Apathy predicts prospective cognitive decline, and not vice versa
- Structural changes in frontal and cingulate regions predict apathy progression
- Apathy is an early marker in frontotemporal dementia, even before dementia onset

**Research in context**

1. **Systematic review**: Previous literature supports the role of apathy in frontotemporal dementia as a disabling feature and risk factor for worse prognosis in terms of survival. However, its role as an early marker and predictor of disease progression remains unclear.
2. **Interpretation**: In presymptomatic carriers of *MAPT*, *GRN*, or *C9orf72* mutations, apathy occurs early, worsens over time, and predicts a subsequent subclinical deterioration of cognitive performance. The progression of apathy is also associated with early brain changes in the frontal lobe and cingulate gyrus. Apathy represents an early marker of cognitive decline and brain changes in presymptomatic frontotemporal dementia.
3. **Future directions**: Apathy assessments in early stages of frontotemporal dementia may improve cohorts’ stratification and future therapeutic trials. Apathy may also be a modifiable factor in its own right, and a target not only for symptomatic treatment but also interventions to slow down or delay clinical decline in people at risk of frontotemporal dementia.

Participants underwent the GENFI standardized assessment. During the first visit, demographic information of all participants, and information regarding clinical background (neuropsychiatric features, family and medical history, medication and symptoms) was collected. The years to the expected symptom onset (EYO) variable for each subject was defined by the mean within each family of affected relatives,^20^ while acknowledging that this is a weak predictor in *GRN* and *C9orf72* families.^31^ Participants underwent a clinical and cognitive assessment to evaluate their symptomatic status and the cognitive performance at the baseline and annually for 2 years. This included structured clinical examination and ratings of behavioral and neuropsychiatric symptoms by clinicians (including sub‐sections of the frontotemporal lobar degeneration clinical dementia rating scale). Behavioral symptoms were assessed using the revised Cambridge Behavioural Inventory (CBI‐R). The neuropsychological battery included tests for language, memory, and executive function. Non‐language based tests relevant to executive function included Digit Span Backwards from the Wechsler Memory Scale‐Revised, Trail Making Test B (TMT B), and the WAIS‐R Digit Symbol Substitution test.^20^ As the measure of apathy severity, we used the motivation subscale of the CBI‐R, which has been used to quantify apathy in previous studies of FTD.^3^, ^7^ This subscale assesses patients’ apathy through their carers’ responses on loss of enthusiasm in personal interests, reduced interest in new things or maintaining social relationships, and indifference to family members. With our main focus on apathy, we excluded subjects without CBI‐R scores across visits (N = 53) from the initial DataFreeze 4 (N = 653). To index executive cognitive deterioration, we used the WAIS‐R Digit Symbol test. This test has high test‐retest reliability,^32^ making it suitable for longitudinal studies. In addition, presymptomatic carriers show reduced performance almost 10 years before their expected age of onset.^20^ We tested the correlation between Digit Symbol scores and two other commonly used executive function related tests, the Digit Span Backwards and TMT B. For each test and analysis, we included z‐scores based on gene‐negative control group data at baseline. The use of z‐scores minimizes the risk of disclosure of genetic status and meets our aim of quantifying the relative severity of symptoms within the cohort, and their covariance with other cognitive and brain measures.

### 2.2 Imaging data acquisition and preprocessing

In DataFreeze 4, 573 out of 600 participants included in this study had at least one volumetric T1‐weighted MRI scan on 3T (or 1.5T scanners at sites where 3T scanning was not available) within 2 years of follow‐up. Magnetization Prepared Rapid Gradient Echo (MPRAGE) images were acquired at each site accommodating different manufacturers and field strengths.^20^ Gray matter regional volumes were extracted from the subcortical segmentation and cortical parcellation labeled by the Desikan‐Killiany Atlas in Freesurfer 6.0 (surfer.nmr.mgh.harvard.edu/). For cases with more than one scan, all available follow‐up images were included in the processing with the longitudinal stream in Freesurfer, creating an unbiased within‐subject template for case‐specific segmentation.^33^ Regional volumes were combined into bilateral frontal, temporal (including amygdala and hippocampus), parietal and occipital lobes, insula cortex, cingulate cortex, subcortical central structures (basal ganglia and thalamus), and brainstem. Carriers’ volumes were z‐scored with reference to non‐carriers. Total intracranial volume (TIV) was estimated as the sum of gray matter, white matter, and cerebrospinal fluid segmentations using the Computational Anatomy Toolbox (CAT12; http://www.neuro.uni-jena.de/cat/) within Statistical Parametric Mapping software (SPM12; http://www.fil.ion.ucl.ac.uk/spm/). CAT12 also provides imaging quality ratings considering noise, motion, and spatial resolution. Raw and parcellated data were visually inspected, and images with significant artifacts, or parcellation failure were excluded, such that all scans included in the analyses had CAT12 imaging quality ratings higher than 74/100 (mean: 84.2, standard deviation: 1.3, range: 74 to 87).

### 2.3 Statistical analyses

#### 2.3.1 Descriptive statistics

Baseline age, education, EYO, CBI‐R apathy scores, and Digit Symbol scores were compared between groups with a two independent‐samples *t* test. Sex was compared between groups with the chi‐square test. Within the two groups, for participants who presented scores > 0 at a depression severity clinical evaluation (0‐3; N = 38 non‐carriers, N = 43 presymptomatic carriers), we tested the baseline association between depression and apathy with the Independent Samples Kruskal‐Wallis Test.

#### 2.3.2 Latent growth curve model

Univariate latent growth curve models (LGCMs) were fitted to the combined data from three time points of longitudinal behavioral/cognitive and imaging assessments, to test the relationships between apathy, cognition, and brain volumes. The LGCM provides insight into baseline scores, change, and individual differences by estimating (1) an intercept, which represents the initial level of the outcome measures; (2) a slope, quantifying the rate of change; (3) a variance of the intercept and slope, capturing individual differences in baseline and change over time; and (4) the relation between intercept and slope, that is, how the initial level is associated with the rate of change over time. Predictors can be added to the model to assess their effects (as an interaction) with intercept and/or slope. The LGCM estimation has two main steps: (1) a linear or curvilinear regression is conducted to fit across the repeated measures of each subject, eliciting a growth curve shape which describes the change over time; and (2) the potential predictors of individual differences in intercepts/slopes are then evaluated. In this way the growth model, as a collection of individual trajectories, describes the individual differences in the changes over time, and the change at group level.^34^ LGCM is a powerful and flexible tool well suited to specifying and testing hypotheses of changes, predictors of change and clinical progression,^34^, ^35^ and can be estimated using open source software such as R (R Core Team). Compared to simpler longitudinal analysis methods, LGCM is preferred for complex models with more than one dependent variable and/or more than one predictor, with complex variance functions, or multigroup model estimation with partial constraints, to assess global model fit, and to deal with random missing data.^35^ LGCM guidelines recommend ≥3 time points and ≥5 cases per parameter.^35^ These requirements were met by our data. Our LGCM were estimated in the lavaan package^36^ using full information maximum likelihood with robust standard errors to deal with missingness and non‐normality. For each model, we considered three main model fit indices^37^: (1) the root‐mean‐square error of approximation (RMSEA, acceptable fit: <0.08, good fit: <0.05), (2) the comparative fit index (CFI, acceptable fit: 0.95‐0.97, good fit: >0.97), and (3) the standardized root mean‐square residual (SRMR, acceptable fit: 0.05‐0.10, good fit: <0.05). We also report the model chi‐square test (χ^2^), noting this index is sensitive to the sample size and is liable to reject models of large cohorts (good fit: low values and *P* > 0.05).^37^ We also report the ratio between chi‐square and degrees of freedom (χ^2^/df) as an alternative model fit index (acceptable fit: <2, good fit: <3).^37^ To test group differences on parameters of interest in LGCMs, we compared each model to a model that constrained the relevant parameters (eg, the slope) to be equal between the two groups. For model comparisons, we used Akaike Information Criteria (AIC), penalizing model complexity.

#### 2.3.3 LGCM of apathy and cognitive decline

In all models, the intercept was centered at baseline and a linear slope was tested. CBI‐R apathy scores and Digit Symbol scores at follow‐up visits were annualized and recomputed at one and 2 years to adjust for small differences in intervals. EYO was included as a predictor of both intercept and slope, and the genetic status used to define groups. We applied four different LGCMs to behavioral and cognitive data to test our main hypothesis: (1) a LGCM on the longitudinal CBI‐R apathy subscale scores; (2) the same as the previous item, but with baseline Digit Symbol as predictor; (3) a LGCM on the longitudinal Digit Symbol scores; and (4) the same as the previous item, but with baseline CBI‐R apathy subscale scores as predictor. These four models allowed us to test whether apathy progresses over time in presymptomatic carriers, and predicts a subclinical cognitive deterioration, or vice versa.

First, an LGCM was fitted on the CBI‐R apathy z‐scores, estimating the parameters freely in a multigroup model defined by genetic diagnosis. This model was compared to one that was fitted by constraining the slope estimation to be equal in the two groups, in order to test the difference in fit of the group equality constrained model with the one accounting for differences between presymptomatic carriers and non‐carriers on the annual rate of change (slope). Second, baseline Digit Symbol scores were added to the model as predictor of both intercept and slope of apathy, to test the predictive value of baseline cognitive performance on longitudinal change in apathy. An analogous approach was applied to the longitudinal and annualized Digit Symbol z‐scores: first, the initial LGCM with EYO as predictor of the intercept and slope was fitted in a multigroup model by freely estimating all parameters; second, we compared this free model with a model where we constrain key parameters to test for between‐group differences; and lastly, baseline CBI‐R apathy scores were added to the model as a predictor variable on intercept and slope.

#### 2.3.4 LGCM for structural brain changes

We applied eight independent univariate LGCMs to estimate longitudinal changes in gray matter volumes of frontal, temporal, parietal and occipital lobes, insular cortex, cingulate cortex, subcortical central structures, and brainstem. As for the behavioral and cognitive scores, all gray matter values at follow‐up visits were computed at 1 and 2 years to adjust for small differences in retest interval. In all models, the intercept was centered at baseline and a linear slope was tested. EYO and TIV were included as predictors of both intercept and slope. Genetic status (presymptomatic carrier versus non‐carrier) defined the groups. When change is homogeneous, or modeled in smaller subgroups, LGCM estimation may occasionally yield improper solutions (ie, impossible values such as negative variances) which necessitate imposing constraints to achieve plausible solutions, which will be noted when necessary. In presymptomatic carriers, we applied a bivariate LGCM model on longitudinal apathy scores and longitudinal gray matter volumes in each of the brain regions that changed over time. With the bivariate LGCM it is possible to investigate the association between the annual rates of change (slopes) in the two variables considered, as well as the associations between initial scores (intercepts) and the longitudinal changes. Thus, we tested our hypothesis on the association between atrophy in fronto‐cingulate brain regions and apathy severity in presymptomatic FTD. For these longitudinal analyses of imaging data, we used an ROI‐based approach that included the regional volumes in the bivariate LGCM with apathy. We considered bilateral lobar values rather than single subregions or lateralized lobar values, to simplify analyses and constrain the parameter‐to‐subject ratio. Although individuals may have asymmetric atrophy, the group pattern is typically bilateral and symmetric. To assess the degree of symmetry, we tested for brain volume differences between left and right hemispheres at baseline using a laterality index (absolute difference between left and right volumes divided by total volume). We then applied *t* tests on this index between presymptomatic carriers and non‐carries, across the whole population and by genetic mutation.

The parameters in each of these models are estimated independently from the other region‐specific models. We correct for multiple comparisons for slope estimates and group comparisons across region‐specific tests, although it would not be appropriate to apply corrections to model fit indices across different models (ie, applying a correction to the chi‐square test of perfect fit would paradoxically improve the apparent fit). We report uncorrected and corrected *P*‐values with False Discovery Rate (FDR) multiple comparisons correction across the eight region‐specific models.

## 3 RESULTS

### 3.1 Descriptive statistics

Demographic and clinical characteristics at baseline, and descriptive statistics are summarized in Table 1. Presymptomatic carriers had higher baseline apathy scores (*P* = 0.015), and were on average 2 years younger (*P* = 0.044) than non‐carriers. At baseline, depression severity and CBI‐R apathy scores were not significantly associated in either non‐carriers (N = 38, range depression scores: 0.5‐3, mean = 1.01, SD = 0.64; Test(3) = 4.134, *P* = 0.247) or presymptomatic carriers (N = 43, range depression scores: 0.5‐2, mean = 1.02, SD = 0.56; Test(2) = 1.129, *P* = 0.569). Depression severity at baseline did not differ between the two groups (χ^2^(3) = 1.79, *P* = 0.618). At baseline, Digit Symbol scores correlated with both TMT B (R = –0.581, *P* < 0.001) and Digit Span Backwards (R = 0.368, *P* < 0.001). In addition, Digit Symbol scores were positively associated with gray matter volumes in frontal lobe (std beta = 0.337, *P* < 0.001, FDR *P* < 0.001), temporal lobe (std beta = 0.231, *P* = 0.0049, FDR *P* = 0.0071), parietal lobe (std beta = 0.280, *P* < 0.001, FDR *P* = 0.00116), occipital lobe (std beta = 0.210, *P* = 0.0016, FDR *P* = 0.0042), cingulate (std beta = 0.205, *P* = 0.0053, FDR *P* = 0.0071), and central structures (std beta = 0.223, *P* = 0.0051, FDR *P* = 0.0071), including TIV and EYO as covariates. However, there was no significant effect of genetic status on this association (presymptomatic carriers vs. non‐carriers; *P* > 0.05).

**TABLE 1:** Demographic and clinical characteristics at baseline for presymptomatic gene carriers and non‐carrier subjects

	Presymptomatic carriers	Non‐carriers	***P*** ‐value
N	304	296	
Age (years; mean ± SD)	44.5 ± 12.1	46.6 ± 14.0	0.044
Sex (Female/Male)	187/117	174/122	0.495
Education (years; mean ± SD)	14.3 ± 3.4	13.9 ± 3.6	0.108
Estimated Years from symptoms Onset (years; mean ± SD)	–14.0 ± 12.1	–13.0 ± 14.1	0.347
CBI‐R Apathy Baseline (z‐scores; mean ± SD)	0.3 ± 1.5	0.0 ± 1.0	0.015
Digit Symbol Baseline (z‐scores; mean ± SD)	0.1 ± 0.9	0.1 ± 1.0	0.948
Total Intracranial Volume Baseline (mean ± SD)	1492.8 ± 142.8	1497.7 ± 141.2	0.684

Uncorrected *P*‐values are the result of *t* test or χ^2^ tests as appropriate: none survive correction for multiple comparisons.

Abbreviation: CBI, Cambridge Behavioural Inventory.

### 3.2 LGCM on longitudinal apathy scores

**FIGURE 1:**
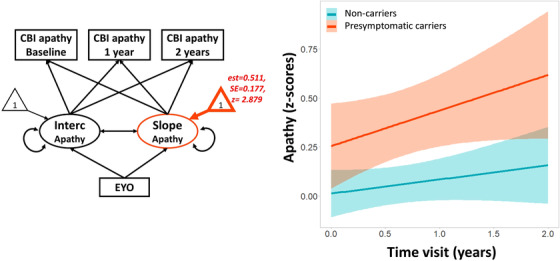
Longitudinal increase in apathy scores over 2‐year period in presymptomatic carriers (red) and non‐carriers (blue). On the left, the latent growth curve model applied to test longitudinal changes in apathy levels, as assessed by the apathy subscale of the revised Cambridge Behavioural Inventory (CBI) over 2 years of follow‐up, including the estimated years from onset (EYO) as covariate. Estimated regression values in presymptomatic group are reported in italics (est = estimate; SE = standard error; z = z‐value). The graph on the right represents apathy scores (y‐axis) at group level over 2‐year follow‐up visits (x‐axis). Individuals’ data are not plotted, to protect anonymity. Abbreviation: Interc, intercept.

**FIGURE A1:**
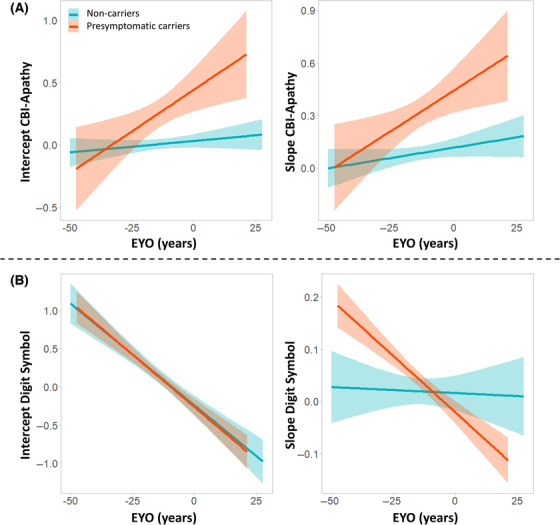
Cross‐sectional and longitudinal changes in apathy severity as assessed by the Cambridge Behavioural Inventory (CBI‐apathy) and Digit Symbol performance in presymptomatic carriers (red) and non‐carriers (blue). (A) Graphs represent the relationships of the estimated initial scores (“intercept”; left graph) and the annual rate of change (“slope”; right graph) in apathy scores with the estimated years from onset (EYO). (B) Graphs represent the relationships of the estimated intercept (left graph) and slope (right graph) in Digit Symbol test scores with EYO. Individuals’ data are not plotted, to protect anonymity.

The LGCM on longitudinal CBI‐R apathy scores fit the data well (χ^2^[11] = 11.59, *P* = 0.395, χ^2^/df = 1.05, RMSEA = 0.025 [0.000‐0.119], CFI = 0.99, SRMR = 0.082), after imposing a necessary constraint (slope variance and intercept‐slope covariance to zero) in non‐carriers. There was a significant increase in apathy scores over time in presymptomatic carriers (estimate [est] = 0.511, standard error (SE) = 0.177, z‐value = 2.879, p = 0.004), but not in non‐carriers (est = 0.084, SE = 0.081, z‐value = 1.036, *P* = 0.300; Figure 1). Comparing the free versus constrained models, the groups differed significantly in the rate of change of apathy (∆χ^2^ ^^= 10.14, ∆df = 1, *P* = 0.0015). EYO was associated with initial values (intercept) of apathy in presymptomatic carriers (est = 0.154, SE = 0.70, z‐value = 2.192, *P* = 0.028) and non‐carriers (est = 0.109, SE = 0.044, z‐value = 2.468, *P* = 0.014) (Appendix, Figure A1, Panel A, left graph), reflecting its association with age in both groups. The effect of EYO on apathy slope in presymptomatic carriers was not significant (est = 0.170, SE = 0.092, z‐value = 1.834, *P* = 0.067; Appendix, Figure A1, Panel A, right graph). Including baseline Digit Symbol scores as a predictor, the model fit the data well (χ^2^[13] = 15.02, *P* = 0.306, χ^2^/df = 1.56, RMSEA = 0.040 [0.000‐0.113], CFI = 0.98, SRMR = 0.073). In presymptomatic carriers, baseline cognitive performance did not influence the rate of change in apathy (est = –0.133, SE = 0.134, z‐value = –0.988, std est = –0.140, *P* = 0.323).

In summary, presymptomatic carriers showed a longitudinal increase in apathy severity over a 2‐year period, which was greater than non‐carriers. This change was not predicted by Digit Symbol test performance at baseline.

### 3.3 LGCM on longitudinal cognition

The LGCM on longitudinal Digit Symbol scores fit the data adequately (χ^2^[11] = 24.68, *P* = 0.010, χ^2^/df = 2.24, RMSEA = 0.079 [0.037‐0.121], CFI = 0.97, SRMR = 0.034), after constraining slope variance and intercept‐slope covariance to zero in non‐carriers. The rate of decline was significant in presymptomatic carriers (est = –0.077, SE = 0.031, z‐value = –2.487, *P* = 0.013), but not in non‐carriers (est = 0.002, SE = 0.023, z‐value = 0.107, *P* = 0.915). Comparing the models confirmed that groups differed significantly in the rate of cognitive decline (∆χ^2^ ^^= 3.912, ∆df = 1, *P* = 0.04796). EYO was associated with initial values (intercept) of Digit Symbol performance in presymptomatic carriers (est = –0.303, SE = 0.038, z‐value = –7.885, *P* < 0.001) and non‐carriers (est = –0.279, SE = 0.039, z‐value = –7.150, *P* < 0.001; Appendix, Figure A1, Panel B, left graph). EYO also modulated the rate of decline in presymptomatic carriers (est = –0.098, SE = 0.024, z‐value = –4.152, *P* < 0.001).

**FIGURE 2:**
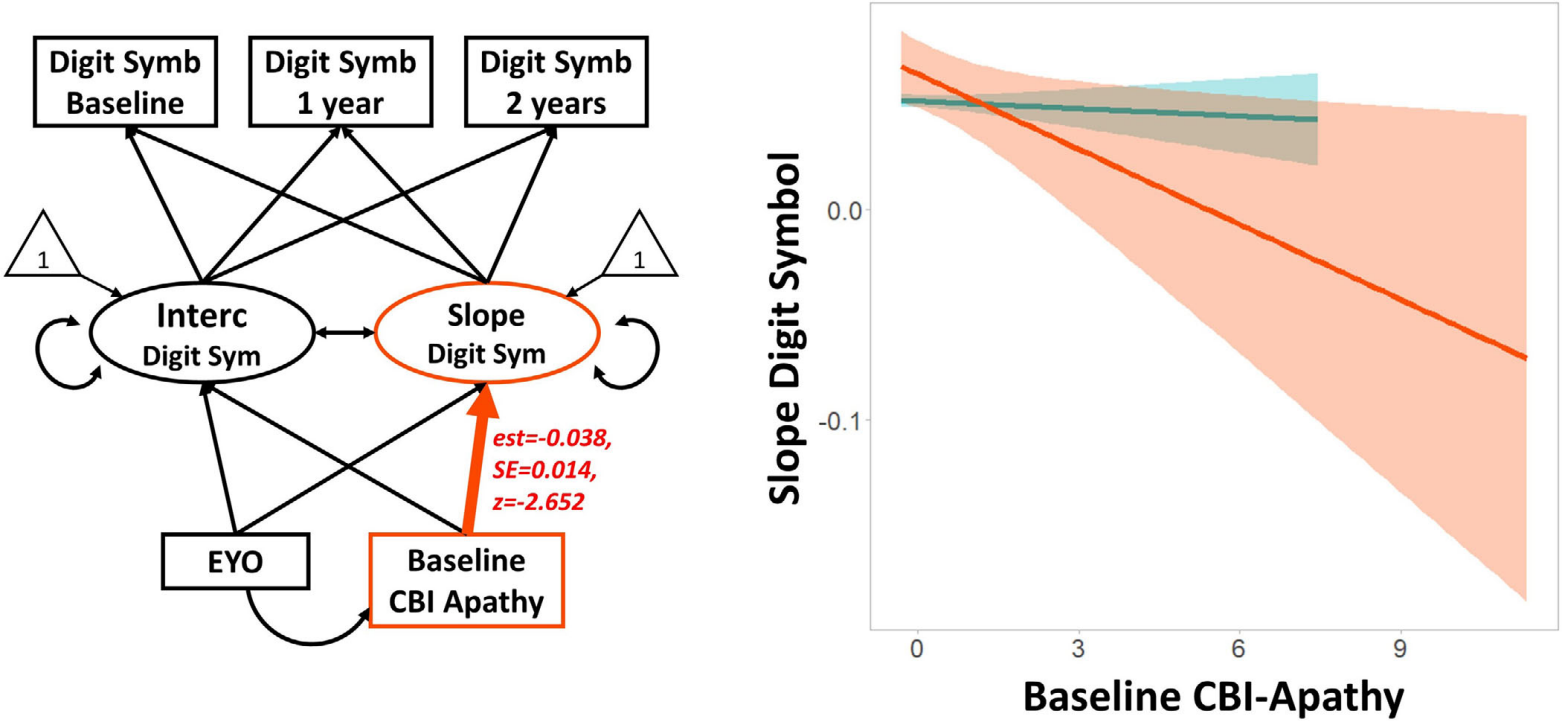
Effect of baseline apathy on the annual rate of change in Digit Symbol performance ('slope'). On the left, the latent growth curve model applied to test the predictive value of baseline apathy levels, as assessed by the apathy subscale of the revised Cambridge Behavioural Inventory (CBI), for longitudinal decline in Digit Symbol test performance over 2 years of follow‐up. The estimated years from onset (EYO) was included as covariate in the model. Estimated regression values in presymptomatic group are reported in italics (est = estimate; SE = standard error; z = z‐value). The graph on the right represents the relationship between the estimated annual rate of change in Digit Symbol performance (y‐axis) and the baseline apathy scores (x‐axis). Individuals’ data are not plotted, to protect anonymity. Abbreviations: Digit Symb, Digit Symbol test; Interc, intercept.

Including baseline CBI‐R apathy scores as a predictor in the model on Digit Symbol longitudinal scores, the model fit the data well (χ^2^[13] = 29.29, *P* = 0.006, χ^2^/df = 2.25, RMSEA = 0.076 [0.039‐0.113], CFI = 0.97, SRMR = 0.030). The model identified a significant decline in presymptomatic carriers only (est = –0.064, SE = 0.031, z‐value = –2.095, *P* = 0.036), with a significant effect of the baseline apathy severity on the rate of cognitive decline (est = –0.038, SE = 0.014, z‐value = –2.652, std est = –0.395, *P* = 0.008; Figure 2), but not on the intercept (est = –0.053, SE = 0.033, z‐value = –1.594, std est = –0.102, *P* = 0.111).

In summary, presymptomatic carriers showed a progressive cognitive decline over 2 years, which was greater than non‐carriers. This subclinical cognitive deterioration was faster when approaching the estimated age of onset, and was predicted by apathy severity at the baseline.

### 3.4 LGCM on longitudinal gray matter brain volumes

Model fit indices for LGCM on z‐scored brain volumes in cortical and subcortical regions, and the estimated slope for both presymptomatic carrier and non‐carrier groups, are reported in Table 2. In summary, for non‐carriers there were no significant structural changes in the regions of interest. In contrast, presymptomatic carriers showed progressive atrophy, which was significantly different from the non‐carrier group, in the frontal, temporal, and parietal lobes, cingulate cortex and in subcortical central structures (but not in the occipital lobe and brainstem). Insular cortex showed longitudinal decline in the presymptomatic group, but this did not significantly differ from non‐carriers’ rate of change. In the model on parietal lobe values, the slope variance term was constrained to zero in non‐carriers to make the model converge correctly. In Appendix B (Table B.1), we also report an exploratory analysis including the gene mutations as a grouping variable in the univariate LGCMs, to estimate longitudinal changes by gene. Group comparisons on annual rates of brain changes indicated differences between gene mutation groups in the temporal lobe and insula. For insula volume, the result was driven by the *C9orf72* group, showing gray matter reduction over time. For temporal lobe volume both *C9orf72* and *MAPT* groups showed a significant annual rate of atrophy, but the *GRN* group did not. However, interpretation of this result requires caution given the unbalanced sample size of the three gene mutation groups.

**TABLE 2:** Model fit indices and estimated slopes of Latent Growth Curve Models on longitudinal z‐scored brain volumes in non‐carriers (Non‐Car) and in presymptomatic carriers (Pres‐Car)

	**Frontal**	**Temporal**	**Parietal**	**Occipital**	**Insula**	**Cingulate**	**Central Structures**	**Brainstem**
**χ** ^2^	24.82	17.01	21.14	15.38	8.56	16.68	18.20	16.21
**χ^2^/df**	2.26	1.55	1.76	1.40	0.78	1.52	1.66	1.47
**RMSEA**	0.068 [0.03‐0.10]	0.049 [0.00‐0.09]	0.058 [0.00‐0.098]	0.041 [0.00‐0.09]	0.00 [0.00‐0.06]	0.043 [0.00‐0.08]	0.053 [0.00‐0.10]	0.048 [0.00‐0.09]
**CFI**	0.99	1.00	0.99	1.00	1.00	1.00	1.00	1.00
**SRMR**	0.013	0.013	0.011	0.013	0.009	0.005	0.004	0.014
**Slope Non‐Car (est, SE, z, *P*)**	–0.015, 0.011, ‐1.381, ***P*** = 0.167	–0.016, 0.011, –1.458, ***P*** = 0.145	0.013, 0.009, 1.434, ***P*** = 0.151	0.014, 0.013, 1.141, ***P*** = 0.254	–0.006, 0.009, –0.676, ***P*** = 0.499	–0.006, 0.006, –0.997, ***P*** = 0.319	–0.010, 0.007, –1.340, ***P*** = 0.180	0.020, 0.011, 1.802, ***P*** = 0.071
**Slope Pres‐Car (est, SE, z, *P*, FDR *P*‐value)**	–0.069, 0.012, –5.907 (*P* < 0.001; < 0.001)*	–0.047, 0.011, –4.459 (*P* < 0.001; < 0.001)*	–0.025, 0.012, –2.148 (*P* = 0.032; 0.043)*	0.017, 0.012, 1.371 (***P*** = 0.170; 0.170)	–0.020, 0.009, –2.156 (*P* = 0.031; 0.043)*	–0.031, 0.007, ‐4.584 (*P* < 0.001; < 0.001)*	–0.052, 0.007, –7.079 (*P* < 0.001; < 0.001)*	0.017, 0.010, 1.713 (***P*** = 0.087; 0.099)
**∆χ** ^2^ **models constraining the slope to equality between groups (*P‐*value; FDR *P*‐value)**	13.61 (*P* = 0.0002; 0.0008)*	4.52 (*P* = 0.034; 0.054)*	6.04 (*P* = 0.014, 0.028)*	0.02 (***P*** = 0.877; 0.877)	1.06 (***P*** = 0.302; 0.403)	8.17 (*P* = 0.004; 0.011)*	28.39 (*P* = 9.94e‐08; 7.95e‐07)*	0.06 ***P*** = (0.806; 0.877)

Abbreviations: CFI, comparative fit index; est, estimate; FDR, false discovery rate correction; RMSEA, root‐mean‐square error of approximation; SRMR, standardized root mean‐square residual; z, z‐value; χ2, chi‐square test.

The group comparisons on the laterality index for each lobar value did not identify asymmetry between left and right volumes in presymptomatic carriers as compared to non‐carrier family members (*P* > 0.05). Considering the gene‐specific groups, only insula showed an effect of laterality (left > right volume, *t* = 2.00; *P* = 0.048) in *MAPT* carriers as compared to the family non‐carrier members.

### 3.5 Bivariate LGCMs on longitudinal apathy scores and gray matter brain volumes

**FIGURE 3:**
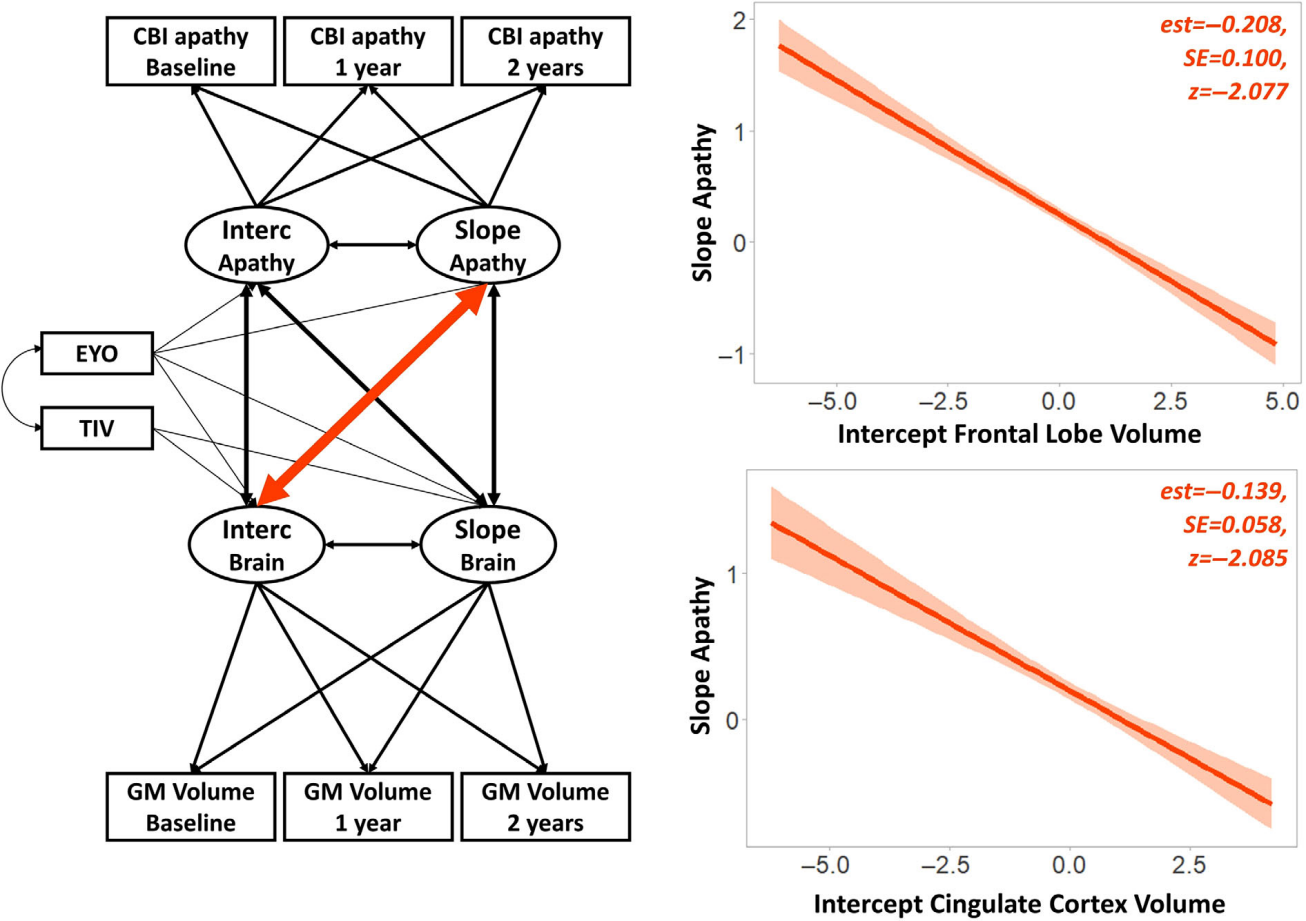
Bivariate latent growth curve model on apathy and gray matter volumes. On the left, the bivariate latent growth curve model (LGCM) applied to test the relationship between longitudinal changes in apathy ('slope'), as assessed by the apathy‐subscale of the revised Cambridge Behavioural Inventory (CBI), and in gray matter (GM) volumes over 2 years of follow‐up. The graphs on the right represent the significant regressions identified by the bivariate LGCMs: annual rate of change in apathy scores (slope, y‐axis) was associated with baseline gray matter volumes in frontal lobe (x‐axis, top graph) and cingulate cortex (x‐axis, bottom graph). Estimated regression values in presymptomatic group are reported in italics (est = estimate; SE = standard error; z = z‐value). Individuals’ data are not plotted, to protect anonymity. Abbreviations: EYO, estimated years from onset; Interc, intercept; TIV, total intracranial volume.

In the previous models of brain changes, significant longitudinal changes in apathy and atrophy were identified in the presymptomatic group only. We therefore applied five new, bivariate, LGCMs of longitudinal apathy and apathy of frontal, temporal, and parietal lobes, cingulate cortex and the subcortical structures, constraining the covariance term between apathy intercept and slope to zero in all models to ensure proper solutions. We report model fit indices and estimated covariance parameters for all brain regions in Table 3. In summary, the annual progression of apathy severity was associated with baseline gray matter volumes in frontal lobe (est = –0.208, SE = 0.100, z = –2.077, std est = –0.348, *P* = 0.038) and cingulate cortex (est = –0.139, SE = 0.058, z = –2.085, std est = –0.237, *P* = 0.037; Figure 3). Comparing the bivariate LGCMs with and without constraining the estimation of covariance between brain volume intercept and progressive apathy to zero, freely estimating the association between brain structure and apathy change improved model fit for both frontal lobe (∆χ^2^ ^^= 5.056, ∆df = 1, *P* = 0.025) and cingulate cortex (∆χ^2^ ^^= 7.206, ∆df = 1, *P* = 0.007) gray matter volumes. With reduced sample sizes in gene specific subgroups, the LGCM method is not suitable for gene‐specific analysis in this dataset. Larger future datasets in GENFI, or merged datasets between genetic FTD cohort studies, may enable gene‐specific modeling.

**TABLE 3:** Model fit indices and estimated covariance parameters of Bivariate Latent Growth Curve Models on longitudinal apathy scores (Ap) and longitudinal brain volumes (Br)

	**Frontal**	**Temporal**	**Parietal**	**Cingulate**	**Central structures**
**χ** ^2^	31.19	36.53	35.10	31.91	31.83
**χ^2^/df**	1.73	2.03	1.95	1.77	1.68
**RMSEA**	0.066 [0.02‐0.10]	0.079 [0.04‐0.12]	0.075 [0.04‐0.11]	0.069 [0.03‐0.11]	0.068 [0.02‐0.11]
**CFI**	0.98	0.97	0.97	0.98	0.98
**SRMR**	0.105	0.111	0.111	0.109	0.114
**Intercept Ap ∼∼ Intercept Br (est, SE, z, *P*)**	–0.067, 0.093, –0.723, *P* = 0.469	0.025, 0.089, 0.280, *P* = 0.780	0.023, 0.062, 0.368, *P* = 0.713	0.037, 0.058, 0.647, *P* = 0.518	0.027, 0.128, 0.210, *P* = 0.834
**Slope Ap ∼∼ Intercept Br (est, SE, z, *P*)**	**–0.208, 0.100, –2.077,** *P* = 0.038*	–0.133, 0.080, –1.662, *P* = 0.097	–0.121, 0.069, –1.735, *P* = 0.083	**–0.139, 0.058, –2.085,** *P* = 0.037*	–0.090, 0.082, ‐1.094, *P* = 0.274
**Slope Br ∼∼ Intercept Ap (est, SE, z, *P*)**	0.002, 0.048, 0.045, *P* = 0.964	–0.018, 0.025, –0.716, *P* = 0.474	0.011, 0.026, 0.424, *P* = 0.672	–0.023, 0.018, –1.304, *P* = 0.192	0.024, 0.030, 0.791, *P* = 0.429
**Slope Ap ∼∼ Slope Br (est, SE, z, *P*)**	–0.003, 0.045, –0.070, *P* = 0.944	0.007, 0.017, 0.447, *P* = 0.655	–0.006, 0.023, –0.268, *P* = 0.789	–0.016, 0.013, –1.176, *P* = 0.240	–0.047, 0.033, –1.435, *P* = 0.151

Abbreviations: Ap, apathy; Br, brain; CFI, comparative fit index; est, estimate; RMSEA, root‐mean‐square error of approximation; SRMR, standardized root mean‐square residual; std est, standard estimate; z, z‐value; χ^2^, chi‐square test.

## 4 DISCUSSION

In this study we found that apathy progresses significantly in presymptomatic carriers of mutations associated with FTD, and that individual differences in apathy at baseline predict the severity of progressive deterioration of performance on the Digit Symbol test over time. In presymptomatic carriers, the progression of apathy over 2 years is associated with atrophy of the frontal lobe and cingulate gyrus at baseline. In contrast, subclinical cognitive impairments do not predict the worsening of apathy.

Apathy is one of the most prevalent symptoms in patients with FTD syndromes,^38^ and is associated with negative outcomes, such as cognitive and functional decline, decreased quality of life, loss of independence and poorer survival.^4^, ^5^ Here we examined the relationship between apathy and cognitive decline over time, through predictive modeling of longitudinal change. Moreover, we did so in the context of the long presymptomatic phase of FTD pathology, lasting many years before dementia onset.^19^, ^20^, ^39^ Among ∼300 carriers, subclinical apathy worsened over 2 years, and was more severe in older carriers. This effect of time was observed with respect to the estimated year of onset of dementia. In contrast, their relatives without mutations did not show emergence of apathy. Carriers showed a similarly faster cognitive deterioration, as assessed with Digit Symbol test performance, before the critical functional threshold that underlines the diagnosis of dementia.^19^, ^20^, ^39^ The rate of cognitive decline was predicted by baseline apathy, but not vice versa, consistent with a predictive effect of apathy on cognitive deterioration, over and above the presence of apathy as an early manifestation of the genetic mutations.

Among the gene carriers, baseline gray matter volume of frontal lobe and cingulate cortex predicted the faster progression of apathy over 2 years. Apathy therefore represents an early neurobehavioral marker that is related to the underlying FTD pathology. Rohrer et al.^20^ reported cross‐sectional atrophy in presymptomatic and symptomatic carriers of mutations in *MAPT*, *GRN*, or *C9orf72*. In relation to estimated year of onset of dementia, there was early volume loss of the insula and temporal lobe (∼10 years before expected symptoms onset), followed by the frontal lobe and subcortical structures (∼5 years before expected onset), parietal and cingulate gyrus (around time of expected onset), and occipital lobe (∼5 years after expected onset). However, cross‐sectional studies are not always predictive of longitudinal changes, and are not informative on causality or the direction of causality. In this larger and longitudinal sample of presymptomatic carriers, the cross‐sectional and longitudinal data are concordant on the progression of apathy and atrophy, and their association in the frontal and cingulate regions.^3^, ^10^, ^11^, ^12^ We pooled our analyses over the pathogenic mutations of *MAPT*, *GRN* and *C9orf72*. The sample size of the genetic subgroups is not sufficient for valid LGCM modeling of separate gene effects, and we cannot use this method with only 304 presymptomatic carrier participants (54 with *MAPT* mutations, 142 with *GRN* mutations and 108 with *C9orf72* mutations) to compare gene effects. There is some evidence of genetic moderation of apathy in dementia, such as the apolipoprotein E gene (*APOE*) ε4 allele in Alzheimer's disease,^40^ as well as *C9orf72* and *GRN* mutations in FTD.^41^ For example, up to 88% of patients with *C9orf72* expansions are reported to develop severe apathy, often as a presenting symptom (see review^42^). Apathy has been reported in ∼69% of patients with *GRN* mutations,^43^ but is less common with *MAPT* mutations.^41^, ^44^, ^45^ Although apathy is sometimes reported as more common than disinhibition,^45^ apathy and disinhibition are strongly positively associated.^10^ Across all three genes, a recent study reported apathy as the most frequent initial symptom in patients with genetic FTD.^46^

There are several limitations to this study. Apathy is a multidimensional construct that is often considered in terms of affective, cognitive, and behavioral components, leading to reduced goal‐directed behaviors. These apathy domains have been identified in patients with FTD, and are associated with lesions or dysfunction involving the fronto‐subcortical networks.^13^, ^47^ We quantified apathy from the subscale of CBI‐R, as it was the principal measure for apathy available in presymptomatic cases from the GENFI study. Although this has been successfully employed in previous studies on FTD, and more recently also in presymptomatic FTD,^46^ this questionnaire is not designed to tease apart the subcomponents of apathy. In addition, as for other carer ratings scales, the emotional distress, personal background, and awareness about the illness may bias the carer's evaluation. However, our results align with evidence in symptomatic FTD patients, showing an early association of apathy reported by patients’ carers with frontal and cingulate gray matter volume degeneration.^13^, ^14^ Similarly, in patients with syndromes of frontotemporal lobar degeneration including FTD, Lansdall et al. reported a significant association of carers’ ratings for apathy with diffuse atrophy in the fronto‐striatum, cingulate, and temporal regions.^3^ Overall these findings suggest a clinicopathological association between apathy severity, as reported by carers, and neurodegeneration in key regions associated with motivation. The GENFI study did not include self‐rated scales for apathy, which rely on insight and introspection abilities that may be lacking in patients with FTD. However, future studies on the presymptomatic phase of FTD may consider also investigating longitudinal changes in self‐rating apathy scores and multimodal apathy assessments (eg, behavioral tests, computer tasks, patient and carer's ratings and questionnaires) to estimate separate domains of the multidimensional construct of apathy, and their associations with cognitive decline.

Executive dysfunction is common in all three genotypes.^20^, ^45^ We quantified cognitive decline with the WAIS‐R Digit Symbol test, which is sensitive to changes in executive function and frontal lobe damage. This test has high test‐retest reliability^32^ and is not a language‐based task, making it well suited to a longitudinal and multinational study like GENFI. The Digit Symbol test involves a range of cognitive operations, not only executive functions but also visuoperceptual scanning and the ability to write or draw.^22^ These are related to visuospatial and motor systems that are not typically impaired in early stages of FTD. However, it does not in itself allow one to dissociate the potential elements of executive cognition, such as selective working memory, inhibitory controls or planning. Given the range of cognitive operations involved in the performance of this test, it might reflect generalized cognitive decline, rather than specific deficits related to executive dysfunction. Nonetheless, in our cohort, Digit Symbol scores correlated strongly with the performance on other executive function tests, and emerged as the most sensitive measure in capturing a subclinical cognitive deterioration in presymptomatic gene carriers.

Another challenge in the quantification of apathy and executive function is the potential overlap with other symptoms, such as depression and akinesia.^38^ In particular, depression might be a confounding symptom for apathy. The wider spectrum of neuropsychiatric symptoms has been described in GENFI, at least in terms of cross‐sectional prevalence of symptoms/signs and their neural correlates.^46^, ^48^ Our study and hypothesis focused on apathy, but we verified that depression and apathy measures were not significantly associated. This suggests that the CBI‐R apathy subscale is not simply measuring, or confounded by, depression symptoms. This aligns with previous evidence that supports the dissociation between apathy and depression in FTD and other neurodegenerative diseases.^15^, ^49^, ^50^, ^51^, ^52^, ^53^ While akinesia is common in symptomatic genetic FTD,^54^ it is not common in presymptomatic cases and does not correlate with apathy measures in other cohorts.^10^ Finally, an open longitudinal study like GENFI will have incomplete longitudinal data. We therefore included only the first three waves of assessment, fulfilling the minimum requirement in the LGCM guidelines.^35^ In years to come, it will be possible to examine a larger data sample and/or a longer follow‐up period, including the role of apathy in the transition from presymptomatic to symptomatic phases of FTD, and the relationship between apathy, cognitive, and brain changes by gene mutation groups.

To conclude, our results demonstrate that apathy occurs early in disease progression of genetic FTD, reflecting early brain changes and predicting individual future clinical trajectories of cognitive and executive function deterioration. The assessment of apathy could help with cohorts’ stratification, according to their prognosis, and improve the power and design of future therapeutic trials. Apathy may also be a modifiable factor in its own right, by pharmacological^55^ or non‐pharmacological interventions.^56^ As such, it becomes a potential target not only for symptomatic treatment but also interventions to slow down or delay clinical decline in people at risk of FTD.

## CONFLICT OF INTEREST

None.

## Supporting information

Supplementary information

## APPENDIX B

### B.1

**TABLE B1:** Model fit indices and estimated slopes of Latent Growth Curve Models on longitudinal z‐scored brain volumes in presymptomatic carriers by gene groups

	**Frontal**	**Temporal**	**Parietal**	**Occipital**	**Insula**	**Cingulate**	**Central Structures**	**Brainstem**
**χ** ^2^	24.13	29.49	39.18	63.70	24.79	20.71	52.74	51.48
**χ^2^/df**	1.42	1.64	2.18	3.54	1.46	1.15	2.93	2.86
**RMSEA**	0.063 [0.00‐0.12]	0.080 [0.02‐0.13]	0.111 [0.06‐0.16]	0.156 [0.12‐0.20]	0.068 [0.00‐0.12]	0.039 [0.00‐0.10]	0.138 [0.096‐0.18]	0.134 [0.09‐0.18]
**CFI**	0.99	0.99	0.98	0.95	0.99	1.00	0.97	0.97
**SRMR**	0.029	0.015	0.018	0.029	0.012	0.008	0.011	0.023
**Slope *C9orf72* (est, SE, z, *P*, FDR *P*‐value)**	**–0.071, 0.016, –4.574 (*P* < 0.001; < 0.001)***	**–0.080, 0.014, –5.696, (*P* < 0.001; < 0.001)***	–0.020, 0.019, –1.036 (***P*** = 0.300; 0.400)	0.018, 0.024, 0.762 (***P*** = 0.446; 0.510)	**–0.067, 0.016, –4.123, (*P* < 0.001;** **< 0.001)***	**–0.043, 0.009, –4.750, (*P* < 0.001;** **< 0.001)***	**–0.073, 0.011, –6.884, (*P* < 0.001;** **< 0.001)***	0.005, 0.016, 0.331 (***P*** = 0.741; 0.741)
**Slope *GRN* (est, SE, z, *P*, FDR *P*‐value)**	**–0.052, 0.016, –3.180 (p = 0.001; 0.004)***	–0.021, 0.014, –1.525 (***P*** = 0.127; 0.162)	–0.022, 0.015, –1.469 (p = 0.142; 0.162)	0.029, 0.017, 1.756 (***P*** = 0.079; 0.130)	0.009, 0.011, 0.812 (***P*** = 0.417; 0.417)	**–0.028, 0.010, –2.931 (*P* = 0.003, 0.008***	**–0.048, 0.011, –4.527, (*P* < 0.001;** **< 0.001)***	0.021, 0.012, 1.744 (***P*** = 0.081; 0.130)
**Slope *MAPT* (est, SE, z, *P*, FDR *P*‐value)**	**–0.113, 0.033, –3.458 (*P* < 0.001;** **< 0.001)***	**–0.088, 0.026, –3.312 (*P* < 0.001;** **< 0.001)***	**–0.069, 0.021, –3.225 (*P* < 0.001;** **< 0.001)***	–0.024, 0.020, –1.183 (***P*** = 0.237; 0.316)	–0.012, 0.026, –0.473. (***P*** = 0.637; 0.637)	–0.026, 0.014, –1.913 (***P*** = 0.056; 0.112)	–0.023, 0.018, –1.259 (***P*** = 0.208; 0.316)	0.021, 0.028, 0.756 (***P*** = 0.450; 0.514)
**∆χ** ^2^ **models constraining the slope to equality between groups (*P* value; FDR *P‐*value)**	2.95 (0.229; 0.305)	**13.89 (0.00096; 0.0068)***	4.29 (0.117; 0.216)	4.03 (0.135; 0.216)	**12.74 (0.0017; 0.0068)***	1.35 (0.509; 0.582)	4.49 (0.106; 0.216)	0.49 (0.745; 0.745)

Abbreviations: CFI, comparative fit index; est, estimate; FDR, false discovery rate correction; Non‐Car, non‐carriers; Pres‐Car, presymptomatic carriers; RMSEA, root‐mean‐square error of approximation; SRMR, standardized root mean‐square residual; z, z‐value; χ2, chi‐square test.
