# Supplementary material for: Apathy in presymptomatic genetic frontotemporal dementia predicts cognitive decline and is driven by structural brain changes
Source: Alzheimers Dement. 2020 Dec 14;17(6):969–83. doi: 10.1002/alz.12252 (PMC8247340; doi:10.1002/alz.12252)
Supplement: Supplementary file 1 — Supplementary information [file ALZ-17-969-s001.docx]

**GENFI consortium collaborators**

Sónia Afonso (Instituto Ciencias Nucleares Aplicadas a Saude, Universidade de Coimbra, Coimbra, Portugal); Maria Rosario Almeida (Faculty of Medicine, University of Coimbra, Coimbra, Portugal)

Sarah Anderl-Straub (Department of Neurology, University of Ulm, Ulm, Germany); Christin Andersson (Department of Clinical Neuroscience, Karolinska Institutet, Stockholm, Sweden); Anna Antonell (Alzheimer’s disease and Other Cognitive Disorders Unit, Neurology Service, Hospital Clínic, Barcelona, Spain); Silvana Archetti (Biotechnology Laboratory, Department of Diagnostics, ASST Brescia Hospital, Brescia, Italy); Andrea Arighi (Fondazione IRCCS Ca’ Granda Ospedale Maggiore Policlinico, Neurodegenerative Diseases Unit, Milan, Italy; University of Milan, Centro Dino Ferrari, Milan, Italy); Mircea Balasa (Alzheimer’s disease and Other Cognitive Disorders Unit, Neurology Service, Hospital Clínic, Barcelona, Spain); Myriam Barandiaran (Cognitive Disorders Unit, Department of Neurology, Donostia University Hospital, San Sebastian, Gipuzkoa, Spain; Neuroscience Area, Biodonostia Health Research Insitute, San Sebastian, Gipuzkoa, Spain); Nuria Bargalló (Imaging Diagnostic Center, Hospital Clínic, Barcelona, Spain); Robart Bartha (Department of Medical Biophysics, The University of Western Ontario, London, Ontario, Canada; Centre for Functional and Metabolic Mapping, Robarts Research Institute, The University of Western Ontario, London, Ontario, Canada); Benjamin Bender (Department of Diagnostic and Interventional Neuroradiology, University of Tübingen, Tübingen, Germany); Alberto Benussi (Centre for Neurodegenerative Disorders, Department of Clinical and Experimental Sciences, University of Brescia, Italy); Luisa Benussi (Istituto di Ricovero e Cura a Carattere Scientifico Istituto Centro San Giovanni di Dio Fatebenefratelli, Brescia, Italy); Valentina Bessi (Department of Neuroscience, Psychology, Drug Research and Child Health, University of Florence, Florence, Italy ); Giuliano Binetti (Istituto di Ricovero e Cura a Carattere Scientifico Istituto Centro San Giovanni di Dio Fatebenefratelli, Brescia, Italy); Sandra Black (Sunnybrook Health Sciences Centre, Sunnybrook Research Institute, University of Toronto, Toronto, Canada); Sergi Borrego-Ecija (Alzheimer’s disease and Other Cognitive Disorders Unit, Neurology Service, Hospital Clínic, Barcelona, Spain); Jose Bras (Dementia Research Institute, Department of Neurodegenerative Disease, UCL Institute of Neurology, Queen Square, London, UK); Rose Bruffaerts (Laboratory for Cognitive Neurology, Department of Neurosciences, KU Leuven, Leuven, Belgium); Marta Cañada (CITA Alzheimer, San Sebastian, Gipuzkoa, Spain); Paola Caroppo (Fondazione IRCCS Istituto Neurologico Carlo Besta, Milano, Italy); Miguel Castelo-Branco (Faculty of Medicine, University of Coimbra, Coimbra, Portugal); Thomas Cope (Department of Clinical Neuriscience, University of Cambridge, Cambridge, UK); Maura Cosseddu (Neurology, ASST Brescia Hospital, Brescia, Italy); Giuseppe Di Fede (Fondazione IRCCS Istituto Neurologico Carlo Besta, Milano, Italy); Alina Díez (Neuroscience Area, Biodonostia Health Research Insitute, San Sebastian, Gipuzkoa, Spain); Diana Duro (Faculty of Medicine, University of Coimbra, Coimbra, Portugal); Chiara Fenoglio (Fondazione IRCCS Ca’ Granda Ospedale Maggiore Policlinico, Neurodegenerative Diseases Unit, Milan, Italy; University of Milan, Centro Dino Ferrari, Milan, Italy); Camilla Ferrari (Department of Neuroscience, Psychology, Drug Research and Child Health, University of Florence, Florence, Italy); Catarina B. Ferreira (Laboratory of Neurosciences, Institute of Molecular Medicine, Faculty of Medicine, University of Lisbon, Lisbon, Portugal); Nick Fox (Department of Neurodegenerative Disease, Dementia Research Centre, UCL Institute of Neurology, Queen Square, London, UK); Morris Freedman (Baycrest Health Sciences, Rotman Research Institute, University of Toronto, Toronto, Canada); Giorgio Fumagalli (Fondazione IRCCS Ca’ Granda Ospedale Maggiore Policlinico, Neurodegenerative Diseases Unit, Milan, Italy; University of Milan, Centro Dino Ferrari, Milan, Italy); Alazne Gabilondo (Neuroscience Area, Biodonostia Health Research Insitute, San Sebastian, Gipuzkoa, Spain); Roberto Gasparotti (Neuroradiology Unit, University of Brescia, Brescia, Italy); Serge Gauthier (Alzheimer Disease Research Unit, McGill Centre for Studies in Aging, Department of Neurology & Neurosurgery, McGill University, Montreal, Québec, Canada); Stefano Gazzina (Neurology, ASST Brescia Hospital, Brescia, Italy); Giorgio Giaccone (Fondazione IRCCS Istituto Neurologico Carlo Besta, Milano, Italy); Ana Gorostidi (Neuroscience Area, Biodonostia Health Research Insitute, San Sebastian, Gipuzkoa, Spain); Caroline Greaves (Department of Neurodegenerative Disease, Dementia Research Centre, UCL Institute of Neurology, Queen Square, London, UK); Rita Guerreiro (Dementia Research Institute, Department of Neurodegenerative Disease, UCL Institute of Neurology, Queen Square, London, UK); Tobias Hoegen (Neurologische Klinik, Ludwig-Maximilians-Universität München, Munich, Germany); Begoña Indakoetxea (Cognitive Disorders Unit, Department of Neurology, Donostia University Hospital, San Sebastian, Gipuzkoa, Spain; Neuroscience Area, Biodonostia Health Research Insitute, San Sebastian, Gipuzkoa, Spain); Vesna Jelic (Division of Clinical Geriatrics, Karolinska Institutet, Stockholm, Sweden); Lize Jiskoot (Department of Neurology, Erasmus Medical Center, Rotterdam, Netherlands); Hans-Otto Karnath (Division of Neuropsychology, Hertie-Institute for Clinical Brain Research and Center of Neurology, University of Tübingen, Tübingen, Germany); Ron Keren (The University Health Network, Toronto Rehabilitation Institute, Toronto, Canada); Tobias Langheinrich (Division of Neuroscience and Experimental Psychology, Wolfson Molecular Imaging Centre, University of Manchester, Manchester, UK); Maria João Leitão (Centre of Neurosciences and Cell Biology, Universidade de Coimbra, Coimbra, Portugal); Albert Lladó (Alzheimer’s disease and Other Cognitive Disorders Unit, Neurology Service, Hospital Clínic, Barcelona, Spain); Gemma Lombardi (Department of Neuroscience, Psychology, Drug Research and Child Health, University of Florence, Florence, Italy); Sandra Loosli (Neurologische Klinik, Ludwig-Maximilians-Universität München, Munich, Germany); Carolina Maruta (Laboratory of Language Research, Centro de Estudos Egas Moniz, Faculty of Medicine, University of Lisbon, Lisbon, Portugal); Simon Mead (MRC Prion Unit, Department of Neurodegenerative Disease, UCL Institute of Neurology, Queen Square, London, UK); Lieke Meeter (Department of Neurology, Erasmus Medical Center, Rotterdam, Netherlands); Gabriel Miltenberger (Faculty of Medicine, University of Lisbon, Lisbon, Portugal); Rick van Minkelen (Department of Clinical Genetics, Erasmus Medical Center, Rotterdam, Netherlands); Sara Mitchell (Sunnybrook Health Sciences Centre, Sunnybrook Research Institute, University of Toronto, Toronto, Canada); Benedetta Nacmias (Department of Neuroscience, Psychology, Drug Research and Child Health, University of Florence, Florence, Italy); Jennifer Nicholas (Department of Medical Statistics, London School of Hygiene and Tropical Medicine, London, UK); Linn Öijerstedt (Center for Alzheimer Research, Division of Neurogeriatrics, Department of Neurobiology, Care Sciences and Society, Bioclinicum, Karolinska Institutet, Solna, Sweden; Unit for Hereditary Dementias, Theme Aging, Karolinska University Hospital, Solna, Sweden); Jaume Olives (Alzheimer’s disease and Other Cognitive Disorders Unit, Neurology Service, Hospital Clínic, Barcelona, Spain); Sebastien Ourselin (School of Biomedical Engineering & Imaging Sciences, King's College London, London, UK); Alessandro Padovani (Centre for Neurodegenerative Disorders, Department of Clinical and Experimental Sciences, University of Brescia, Italy); Jessica Panman (Department of Neurology, Erasmus Medical Center, Rotterdam, Netherlands); Janne Papma (Department of Neurology, Erasmus Medical Center, Rotterdam); Michela Pievani (Istituto di Ricovero e Cura a Carattere Scientifico Istituto Centro San Giovanni di Dio Fatebenefratelli, Brescia, Italy); Yolande Pijnenburg (Amsterdam University Medical Centre, Amsterdam VUmc, Amsterdam, Netherlands); Cristina Polito (Department of Biomedical, Experimental and Clinical Sciences “Mario Serio”, Nuclear Medicine Unit, University of Florence, Florence, Italy); Enrico Premi (Stroke Unit, ASST Brescia Hospital, Brescia, Italy); Sara Prioni (Fondazione IRCCS Istituto Neurologico Carlo Besta, Milano, Italy); Catharina Prix (Neurologische Klinik, Ludwig-Maximilians-Universität München, Munich, Germany); Rosa Rademakers (Department of Neurosciences, Mayo Clinic, Jacksonville, Florida, USA); Veronica Redaelli (Fondazione IRCCS Istituto Neurologico Carlo Besta, Milano, Italy); Ekaterina Rogaeva (Tanz Centre for Research in Neurodegenerative Diseases, University of Toronto, Toronto, Canada); Pedro Rosa-Neto (Translational Neuroimaging Laboratory, McGill Centre for Studies in Aging, McGill University, Montreal, Québec, Canada); Giacomina Rossi (Fondazione IRCCS Istituto Neurologico Carlo Besta, Milano, Italy); Martin Rosser (Department of Neurodegenerative Disease, Dementia Research Centre, UCL Institute of Neurology, Queen Square, London, UK); Beatriz Santiago (Neurology Department, Centro Hospitalar e Universitario de Coimbra, Coimbra, Portugal); Elio Scarpini (Fondazione IRCCS Ca’ Granda Ospedale Maggiore Policlinico, Neurodegenerative Diseases Unit, Milan, Italy; University of Milan, Centro Dino Ferrari, Milan, Italy); Sonja Schönecker (Neurologische Klinik, Ludwig-Maximilians-Universität München, Munich, Germany); Elisa Semler (Department of Neurology, University of Ulm, Ulm); Rachelle Shafei (Department of Neurodegenerative Disease, Dementia Research Centre, UCL Institute of Neurology, Queen Square, London, UK); Christen Shoesmith (Department of Clinical Neurological Sciences, University of Western Ontario, London, Ontario, Canada); Miguel Tábuas-Pereira (Neurology Department, Centro Hospitalar e Universitario de Coimbra, Coimbra, Portugal); Mikel Tainta (Neuroscience Area, Biodonostia Health Research Insitute, San Sebastian, Gipuzkoa, Spain); Ricardo Taipa (Neuropathology Unit and Department of Neurology, Centro Hospitalar do Porto - Hospital de Santo António, Oporto, Portugal); David Tang-Wai (The University Health Network, Krembil Research Institute, Toronto, Canada); David L Thomas (Neuroimaging Analysis Centre, Department of Brain Repair and Rehabilitation, UCL Institute of Neurology, Queen Square, London, UK); Paul Thompson (Division of Neuroscience and Experimental Psychology, Wolfson Molecular Imaging Centre, University of Manchester, Manchester, UK); Hakan Thonberg (Center for Alzheimer Research, Division of Neurogeriatrics, Karolinska Institutet, Stockholm, Sweden); Carolyn Timberlake (Department of Clinical Neurosciences, University of Cambridge, Cambridge, UK); Pietro Tiraboschi (Fondazione IRCCS Istituto Neurologico Carlo Besta, Milano, Italy); Philip Van Damme (Neurology Service, University Hospitals Leuven, Belgium; Laboratory for Neurobiology, VIB-KU Leuven Centre for Brain Research, Leuven, Belgium); Mathieu Vandenbulcke (Geriatric Psychiatry Service, University Hospitals Leuven, Belgium; Neuropsychiatry, Department of Neurosciences, KU Leuven, Leuven, Belgium); Michele Veldsman (Nuffield Department of Clinical Neurosciences, Medical Sciences Division, University of Oxford, Oxford, UK); Ana Verdelho (Department of Neurosciences and Mental Health, Centro Hospitalar Lisboa Norte - Hospital de Santa Maria & Faculty of Medicine, University of Lisbon, Lisbon, Portugal); Jorge Villanua (OSATEK, University of Donostia, San Sebastian, Gipuzkoa, Spain); Jason Warren (Department of Neurodegenerative Disease, Dementia Research Centre, UCL Institute of Neurology, Queen Square, London, UK); Carlo Wilke (Department of Neurodegenerative Diseases, Hertie-Institute for Clinical Brain Research and Center of Neurology, University of Tübingen, Tübingen, Germany; Center for Neurodegenerative Diseases (DZNE), Tübingen, Germany); Ione Woollacott (Department of Neurodegenerative Disease, Dementia Research Centre, UCL Institute of Neurology, Queen Square, London, UK); Elisabeth Wlasich (Neurologische Klinik, Ludwig-Maximilians-Universität München, Munich, Germany); Henrik Zetterberg (Dementia Research Institute, Department of Neurodegenerative Disease, UCL Institute of Neurology, Queen Square, London, UK); Miren Zulaica (Neuroscience Area, Biodonostia Health Research Insitute, San Sebastian, Gipuzkoa, Spain)
